# Supplementary material for: Artificial intelligence–Driven detection and decision support system for precision management of maize downy mildew
Source: PLoS One. 2026 Mar 9;21(3):e0343517. doi: 10.1371/journal.pone.0343517 (PMC12970884; doi:10.1371/journal.pone.0343517)
Supplement: Attachment — Submitted filename: renamed_9edbd.pdf [file pone.0343517.s001.pdf]

**Title: Artificial Intelligence-Driven Detection and Decision Support System for Precision Management of Maize Downy Mildew.**

**Overall Assessment**

This manuscript presents a comprehensive and highly valuable study on developing and validating an integrated Artificial Intelligence-driven system for the detection and management of Maize Downy Mildew (MDM). The work is commendable for its rigorous comparison of thirteen Machine Learning (ML) and Deep Learning (DL) models and its inclusion of advanced interpretability analyses (t-SNE and Grad-CAM). The paper successfully extends its contribution beyond model development by creating a functional web-based Decision Support System (DSS) and validating its associated advisory measures through two years of field trials. The superior performance of the VGG16 model (97.0% accuracy, 0.99 AUC-ROC) and the significant positive impact of the DSS-guided fungicide applications (93-96% disease reduction, 195-289% yield increase) demonstrate the practical relevance of this research. The manuscript is generally well-written and logically structured. However, the following requires clarification or correction:

**Comments**

**1.** There is a significant inconsistency in the description and reported size of the testing dataset, which is critical for verifying the reported model performance.

- The total dataset is stated to be **6252 images** (3100 healthy + 3152 diseased).
- The partitioning is first stated as a **70:15:15 split** for training, validation, and testing. A 15% test set would be approximately **938 samples**.
- A subsequent sentence adjusts this, stating the split is **70% training, 15% validation, and 10% test**. A 10% test set would be approximately **625 samples**.
- The confusion matrix results (in the *Results* section) report a total of 156 healthy + 117 infected = 273 samples for the final evaluation. This number is closer to 4.4% of the total dataset, which contradicts both the 10% and 15% split ratios.

The authors must explicitly state the final, correct partitioning ratio (e.g., 70:15:15, 75:15:10, or 90:5:5) and confirm the exact number of images in the final, independent test set used for the reported metrics. This correction should be consistent between the Methods and Results sections.

**2.** The authors apply regression-based metrics (MAE, MSE, RMSE, $R^2$, MBD) to evaluate the VGG16 model, which is a binary classification model (Healthy vs. Diseased). The authors should justify this approach in the **Methods** section. Specifically, the authors should explain **what continuous values were used for "predicted" and "actual" outputs?** Were the labels (e.g., Healthy=0, Diseased=1) treated as continuous variables, or were the model's output probability scores (e.g., the confidence score for the "Diseased" class) used as the predicted value?

**3. One of the** main contributions of this paper is the integration of the AI model into a Decision Support System and the validation of its advisory measures. The Methods section (V. Avoidable Yield Loss...) describes a standard field trial comparing two treatments: a specific fungicide combination (seed treatment + foliar spray) and an untreated control (UTC). However, the description does **not** specify *how* the DSS was used to **guide** the application. Did the DSS recommend: (1) **When** to apply the foliar spray (based on image data/model output)? (2) **Which** plots or sections to apply the spray to (precision agriculture)? The current methods merely validate the effectiveness of the fungicide chemical itself, which is not the same as validating a decision support system.

**4.** The authors should expand the **Methods** section (Section V.) to detail the actual protocol for the "DSS-guided fungicide applications". If the DSS only provided the chemical *recommendation* which was then applied uniformly, the claims about validating the *Decision Support System* need to be rephrased to accurately reflect what was tested.

**5.** The manuscript states that thirteen ML and DL algorithms were evaluated, and the results section briefly discusses the performance of a few (Logistic Regression, KNN, DT, SVM, GBM, RF, RNN, LSTM, DBN, CNN, Siamese Networks, and DLTL). To enable a complete peer review and maximize the value of the comparative study, a **comprehensive table** should be included that lists all performance metrics (Accuracy, Precision, Recall, F1-Score, AUC-ROC) for all thirteen models.

**6.** The citation for regression metrics is referred to as Jadesha et al. [25a]. Please ensure this citation adheres to the journal's standard numerical or author-year format, as the [25a] suffix may indicate a non-standard reference convention.
